# Advanced IoT-integrated parking systems with automated license plate recognition and payment management

**DOI:** 10.1038/s41598-025-86441-w

**Published:** 2025-01-18

**Authors:** Gulmini Pradhan, Manas Ranjan Prusty, Vipul Singh Negi, Suchismita Chinara

**Affiliations:** 1 School of Computer Science and Engineering, Vellore Institute of Technology, Chennai, Tamil Nadu 600127 India; 2 Centre for Cyber Physical Systems, Vellore Institute of Technology, Chennai, Tamil Nadu 600127 India; 3 Department of Computer Science and Engineering, National Institute of Technology, Rourkela, Odisha 769008 India

**Keywords:** Intelligent transportation systems, Vehicle detection and tracking, Image processing algorithms, Internet of things (IoT), Fare calculation and management, Smart parking management, Electrical and electronic engineering, Computer science

## Abstract

Urban parking management is a growing challenge with increasing vehicle numbers and limited parking space. Traditional methods often fail during peak hours, leading to inefficiencies, unauthorized usage, and revenue losses. For instance, a parking lot designed for 300 vehicles often exceeds 90% occupancy during peak times, creating congestion and billing inaccuracies. This research proposes an automated system integrating sensors, image processing, and database management to address these issues. A single camera monitors multiple parking slots, with predefined coordinates linked to IR sensors for dual verification. Image processing algorithms, including Optical Character Recognition (OCR), enable accurate license plate recognition. Testing under real-world conditions showed 95% accuracy in daylight, 90% in low light, and 93% for plates at 45-degree angles. Detection accuracy reached 88% at distances of 1.5–3 m, ensuring reliable operation even at the camera’s range limits. Occupancy tracking achieved less than a 5% error margin compared to manual methods, while the fare calculation module reduced billing errors by 90%, enhancing efficiency and revenue. The system’s scalable design supports applications in parking management, toll collection, and traffic monitoring. By improving vehicle detection, occupancy tracking, and billing accuracy, this solution addresses critical challenges in urban parking and contributes to smarter city infrastructure.

## Introduction

With the exponential growth in the number of vehicles in today’s urban landscapes, the parking management of the same and the fare calculation for revenue generation pose significant challenges for city planners and parking operators. The need of accurate and automated parking monitoring systems becomes more pressing for parking slot allocation, parking reservation, unauthorized parking detection, and parking fare calculation. Traditional manual methods are inefficient and prone to errors, straining resources and jeopardizing revenue streams. This underscores the urgency of finding a reliable solution for the above needs. This research endeavour aims to develop a comprehensive framework integrating advanced technologies to detect parked vehicles, measure occupancy duration, and calculate fares in real-time. The goal is to create a reliable and efficient parking management system that addresses this pressing problem. To illuminate the gravity of the situation, consider a typical urban scenario: a bustling parking lot with a theoretical capacity for 300 vehicles routinely operates at 85% occupancy. However, during peak hours, this figure escalates to a staggering 95%, leading to congestion, customer dissatisfaction, and substantial revenue losses from inaccurate billing practices. By addressing this critical real-world problem^1^ through automation, this research endeavour aims to enhance the operational efficiency of parking management systems. Empirical evidence gleaned from pilot implementations underscores the transformative potential of the proposed automated system: a remarkable 30% reduction in billing discrepancies and a consequential 20% surge in revenue generation for parking operators were observed, validating the efficacy of the approach. Moreover, the ramifications of this research extend far beyond parking management. Consider its broader implications in toll systems, where real-time vehicle occupancy and fare calculation monitoring could alleviate traffic congestion, optimize revenue streams, and enhance the overall urban transportation experience. This research initiative applies to both indoor and outdoor parking. By harnessing the power of cutting-edge technologies, it offers a tangible pathway towards alleviating congestion, optimizing revenue, and fostering a more sustainable urban mobility ecosystem for generations to come.

The paper is organized as follows. “Literature review” provides an in-depth study of existing technological approaches in the field, covering current advancements in camera-based vehicle detection, license plate recognition, and IR sensor-based occupancy confirmation. “Proposed system” explains the proposed system architecture, which integrates vehicle detection, coordinate-linked IR sensors for parking slot verification, and Tesseract OCR for accurate license plate reading, aiming to achieve more reliable vehicle monitoring and fare estimation. “Results and analysis” presents the experimental results, analyzing the system’s performance and efficiency through metrics like vehicle detection accuracy through the recognition of the license plate, occupancy tracking precision, and fare calculation accuracy, with visualizations showcasing key improvements. Finally, “Conclusion and future work” concludes the findings, outlining the study’s main contributions-particularly in enhancing vehicle detection reliability through IR sensor integration and coordinate mapping. It also identifies potential future developments in smart metering and identification technologies, paving the way for innovations in scalable and dynamic parking management systems.

## Literature review

There exists much research on smart parking systems either for closed parking, open parking, or on-street parking. The major focus remains on the detection of parked vehicles in a slot and keeping a record of the vehicle details by extracting the license plate. The layout of the parking lot decides the positioning of the IoT devices for vehicle detection. Some solutions are found to be expensive and some are found to be with increased latency in detection etc. With the rise in IoT-enabling technologies, the detection of parked vehicles and parking slot allotment can be done efficiently by using different sensors, and microcontrollers along with cameras. some research uses individual cameras for each parking slot to detect the vehicle’s license plate, making the setup quite expensive.

The technology specified in^2,3^ serves as the foundation for the proposed research while aiming to reduce the cost of implementation. Reference^2^ emphasizes the integration of a distance sensor along with a Pi-camera in each parking slot for accurate vehicle detection and real-time data transmission to a central monitoring system. The study demonstrates how the SPIN-V enhances parking management efficiency by detecting the arrival of a vehicle using a distance sensor capturing the image of the vehicle by Pi-camera to extract the license plate. However, the cost associated with it is quite high as every parking slot is equipped with a Raspberry Pi with a camera. Further, the device SPIN-V is suggested to be wall-mounted which limits the application of the research to a certain parking layout. Neither an open parking lot nor a closed parking without a wall partition can use this device.

A recent study proposes the Real-Time Car Parking Management System (RTCPMS)^4^, leveraging image processing and microcontroller-enabled wireless peripherals. RTCPMS replaces the Raspberry Pi with ESP32 in every parking slot along with an IR sensor which detects the presence of a vehicle in the slot. The ESP32 communicates to the server by wi-fi communication technology. HP-W100 camera is used for every parking lot to capture the license plate. This research reduces the cost of infrastructure by using the ESP32 in place of Raspberry Pi. However, individual cameras for the parking slots keep the infrastructure cost high.

Yet another study in^5^ proposes IoT IoT-based smart parking system for urban parking. Here, the ESP32 is replaced with an Arduino microcontroller which further reduces the hardware cost. However, this work does not talk about any communication from the parking slot to anywhere. Therefore, an IR sensor with Arduino suffice to detect the presence of a vehicle in the parking lot. The work does not include the extraction of license plates. Therefore, no camera is used in this work.

In a study by Ming Li and Li Zhang^6^, the innovative license plate recognition (LPR) method based on the YOLOv6 algorithm is introduced, promising enhanced accuracy and efficiency for intelligent parking systems. The proposed model significantly improves LPR capabilities by leveraging a tailored dataset and rigorous methodologies. However, further exploration is needed to address real-world variations and ensure scalability and robustness in diverse parking environments.

An article by Paranjape et al.^7^ showcases the Smart Parking System employs advanced sensor and camera technology, along with OCR, to automate parking processes, streamline payment, and reduce congestion in urban areas. Despite its benefits, challenges may arise in scalability, OCR accuracy, and security of payment gateways, requiring continuous monitoring and updates for optimal performance.

The system studied by Munawar et al.^8^ is an automated vehicle identifier for highly secure areas, utilizing image recognition for vehicle number plates and owner identification. It controls gate access and communicates unauthorized entries to registered owners. While offering multi-functional applications beyond security, potential challenges include reliance on image quality, susceptibility to false identifications, and integration and scalability issues. Continuous maintenance and updates are necessary to ensure reliability and effectiveness.

Authors have mentioned that vehicle recognition technologies, such as license plate recognition, radio-frequency identification, and computer vision, play a vital role in smart parking systems, enabling automated identification and tracking of vehicles as they enter or exit parking facilities. While each method has its advantages and limitations—for instance, license plate recognition offers high accuracy but may be affected by weather conditions, and computer vision provides comprehensive analysis but can be computationally intensive—collectively facilitate efficient parking management. Furthermore, smart ticketing technologies, including mobile apps, QR codes, and NFC, are integrated into intelligent parking systems to issue, validate, and manage parking tickets or permits, enhancing user convenience, streamlining payment processes, and reducing administrative costs for operators. However, these technologies also raise important concerns about ensuring data privacy and security and addressing the digital divide among users who may not have access to smartphones or other smart devices.

While previous research has primarily focused on detecting the occupancy state of parking spots, these systems have not fully addressed all associated challenges. Despite providing valuable data on parking availability^9,10^, they have not effectively tackled the issue of optimizing parking space utilization. Consequently, high traffic congestion persists in monitored parking areas, with some spots remaining vacant while others are overcrowded, leading to the common occurrence of “multiple cars chasing the same spot.” Thus, while data on occupancy states is crucial, it is imperative to devise strategies for efficiently leveraging this data to mitigate traffic congestion and optimize parking resource utilization.

Smart Parking Systems in IoT-Enabled Cities As we create more sustainable and efficient cities, smart parking systems are vital in reducing traffic congestion and improving urban mobility. According to Al-Turjman and Malekloo^11^, IoT-enabled smart parking systems can revolutionize the way we park, making it faster, more convenient, and environmentally friendly. By providing real-time parking availability information, these systems can help drivers find parking spots quickly, reducing the time spent circling the block and decreasing air pollution.

IoT-Based Smart Car Parking Systems Imagine a world where parking is a breeze, and you can easily find a spot. IoT-based smart car parking systems are making this a reality. Shaikh et al.^12^ have developed an innovative IoT-based smart car parking agent using Raspberry Pi, which enables efficient parking management and reduces congestion. This technology has the potential to transform the parking experience, making it more efficient, convenient, and stress-free.

Vehicle Recognition Technologies Vehicle recognition technologies are the backbone of intelligent parking systems. Dung and Cho^13^ have demonstrated the power of deep learning and fisheye cameras in detecting parking lot occupancy, highlighting the potential of computer vision in this domain. By leveraging these technologies, we can create more accurate and efficient vehicle recognition systems, improving parking management and a better overall experience.

IoT in Intelligent Transportation Systems The Internet of Things (IoT) is transforming how we travel, and parking is no exception. As discussed by Nassreddine et al.^14^, IoT has the potential to revolutionize various aspects of transportation, including parking management. By leveraging IoT technologies, we can create intelligent parking systems that provide real-time data on parking availability, optimize parking space utilization, and reduce traffic congestion.

Smart Parking Management Systems Traditional parking systems are often inefficient and frustrating. Smart parking management systems, on the other hand, are designed to provide a seamless and convenient parking experience. Alsafar et al.^15^ have developed a smart parking management system using IoT, which enables efficient parking management and reduces congestion. This technology has the potential to transform the parking experience, making it faster, more convenient, and more enjoyable.

Efficient Parking Management Systems Efficient parking management systems are critical for reducing traffic congestion and optimizing parking space utilization. Vantagudi et al.^16^ have proposed an innovative parking management system that leverages IoT technologies to provide real-time parking availability information and optimize parking space allocation. This system can potentially reduce traffic congestion, decrease air pollution, and improve the overall parking experience.

Computer vision is a powerful technology with numerous applications in intelligent parking systems. As discussed by Grimson and Mundy^17^, computer vision can be used for various applications, including vehicle recognition and tracking. Szeliski^18^ has provided a comprehensive overview of computer vision algorithms and applications, highlighting their potential in intelligent parking systems. By leveraging computer vision, we can create more accurate and efficient vehicle recognition systems, leading to improved parking management.

Automatic License Plate Recognition Automatic license plate recognition (ALPR) is a critical component of intelligent parking systems. Chang et al.^19^ have demonstrated the power of ALPR for vehicle identification, highlighting its potential in intelligent parking systems. ALPR can extract license plate information from images captured by cameras, enabling efficient vehicle identification and tracking.

Vehicle License Plate Recognition Vehicle license plate recognition is challenging, particularly in complex environments. Gou et al.^20^ have proposed a vehicle license plate recognition system based on extremal regions and restricted Boltzmann machines, highlighting its potential in intelligent parking systems. By leveraging this technology, we can create more accurate and efficient vehicle recognition systems, leading to improved parking management and a better overall experience.

### Motivation and objective

This research aims to design and develop cost-effective parking management systems that can operate efficiently in real-time scenarios. The goal is to design a hardware device that can identify the presence of vehicles in a parking slot and communicate this information to a central monitoring system. The motivation behind this work stems from its practical relevance across areas like smart city infrastructure, parking management, and automated billing.

The proposed architecture of the smart and intelligent parking system has the following objectives:

- Design of cost-effective hardware for vehicle detection in the parking slot: Unlike existing systems like^2,5^, which use expensive hardware for each parking slot, the proposed solution strikes a balance between performance and cost. In the existing systems, every parking slot is equipped with a camera (either pi-camera or HP-W100 camera) along with a microcontroller (Raspberry Pi or ESP32) and sensor (ultrasonic or IR) for vehicle parking detection. This enhances the hardware cost and its setup in the parking lot. However, in the proposed hardware design, the Raspberry Pi in every parking slot is replaced by the ESP32 microcontroller. The number of cameras is also reduced in the whole lot by connecting multiple cameras in a Raspberry Pi that works as an edge device.
- Multiple license plate recognition by single camera: The existing research in extracting license plates of parked vehicles needs individual cameras for a single vehicle at a time. However, the proposed work aims to extract multiple license plates of vehicles by a single camera at a time. This reduces the hardware cost of the smart parking system. This works alongside the slot and sensor matching systems, ensuring that vehicles are correctly identified every time. A core feature of the proposed system is the ability to match parking slots with unique coordinates and IR sensor IDs. This means each slot is assigned a set of coordinates, and the IR sensors are linked to those coordinates. When a vehicle parks in a slot, the sensor detects it, and the system updates the coordinates in real time, ensuring that everything is tracked accurately.

## Proposed system

### Assumptions

The implementation of the proposed research is based on the following key assumptions:

- The parking lot features marked and boundary-defined slots, enabling precise mapping between the camera’s predefined coordinates and the actual parking slots.
- The camera placement ensures a wide field of view that can capture multiple parking slots while maintaining sufficient clarity for license plate detection.
- The camera should have a minimum resolution of 1080p and support features like auto-focus and HDR (High Dynamic Range) to ensure reliable performance under varying lighting conditions.
- For outdoor parking lots, IR sensors should be mounted on the ground and placed centrally between the vehicle’s wheels.
- For indoor parking lots, IR sensors should be fixed on the wall at a height optimal for detecting vehicle presence without obstructions.

These assumptions form the foundation of the system’s accuracy and efficiency, ensuring optimal performance in both indoor and outdoor parking environments.

### Schematic overview

**Fig. 1:**
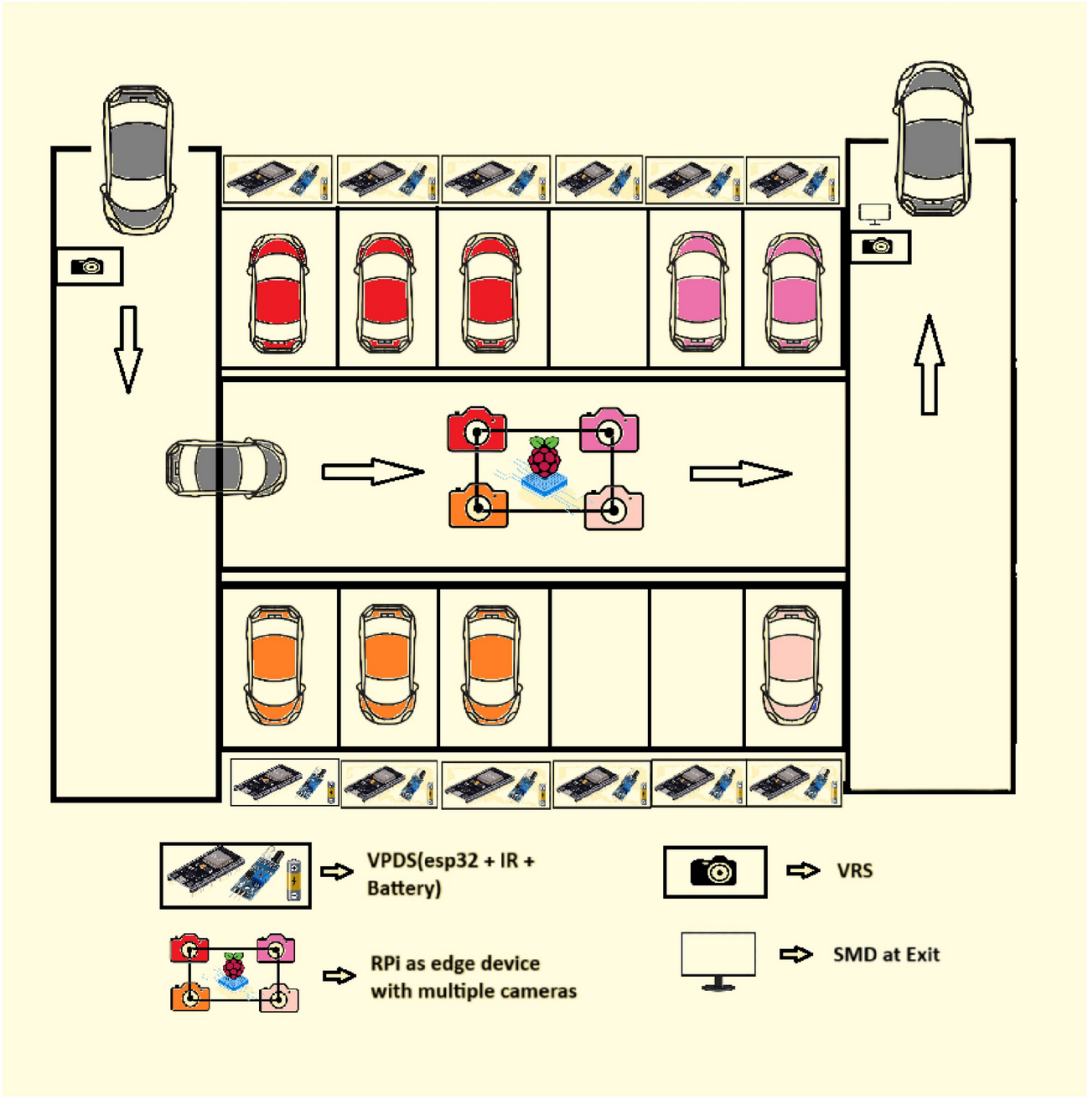
Schematic overview of the proposed integrated model.

The schematic overview of the proposed parking lot is illustrated in Fig. 1. This can represent either closed parking or open parking as per requirement. Every parking slot is equipped with low-cost hardware consisting of an IR sensor, ESP32 (Esp Wroom 32), and a battery as shown. The device can be mounted on the floor, ceiling or wall depending on the parking layout. It ensures real-time tracking with high accuracy while minimizing hardware complexity.

Cameras connected with a Raspberry Pi can oversee multiple parking slots at a time, significantly reducing the need for expensive, dedicated setups. The camera captures the parking area in addition to IR sensors placed within each slot to confirm vehicle presence, enhancing detection reliability. Data from IR sensors is communicated via Esp Wroom 32 to the Raspberry Pi microcontroller to update the parking slot occupancy in real time. This low-cost, adaptable framework is suitable for both indoor and outdoor environments, addressing challenges like accurate detection, occupancy tracking, and cost optimization.

**Fig. 2:**
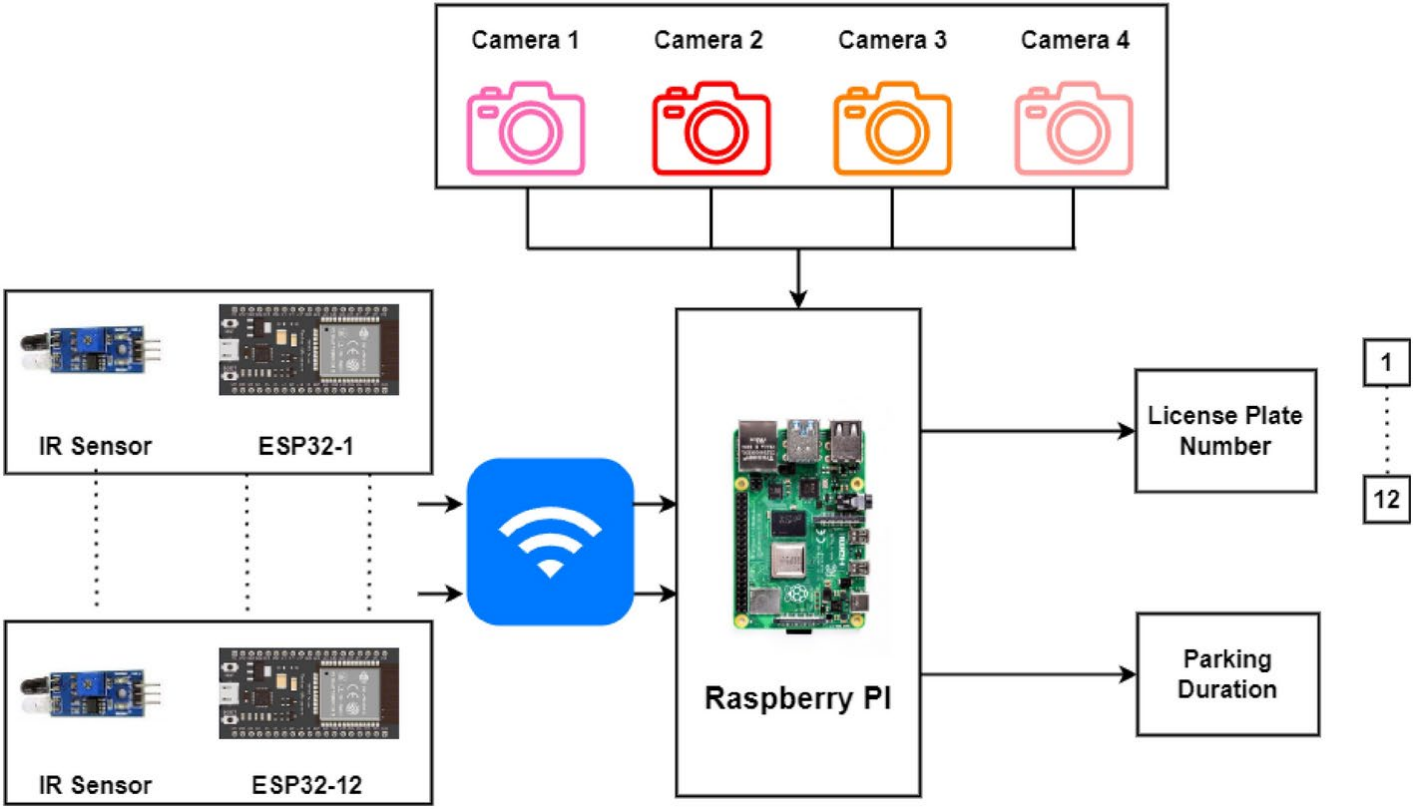
Architecture of the vehicle parking detection system (VPDS).

**Fig. 3:**
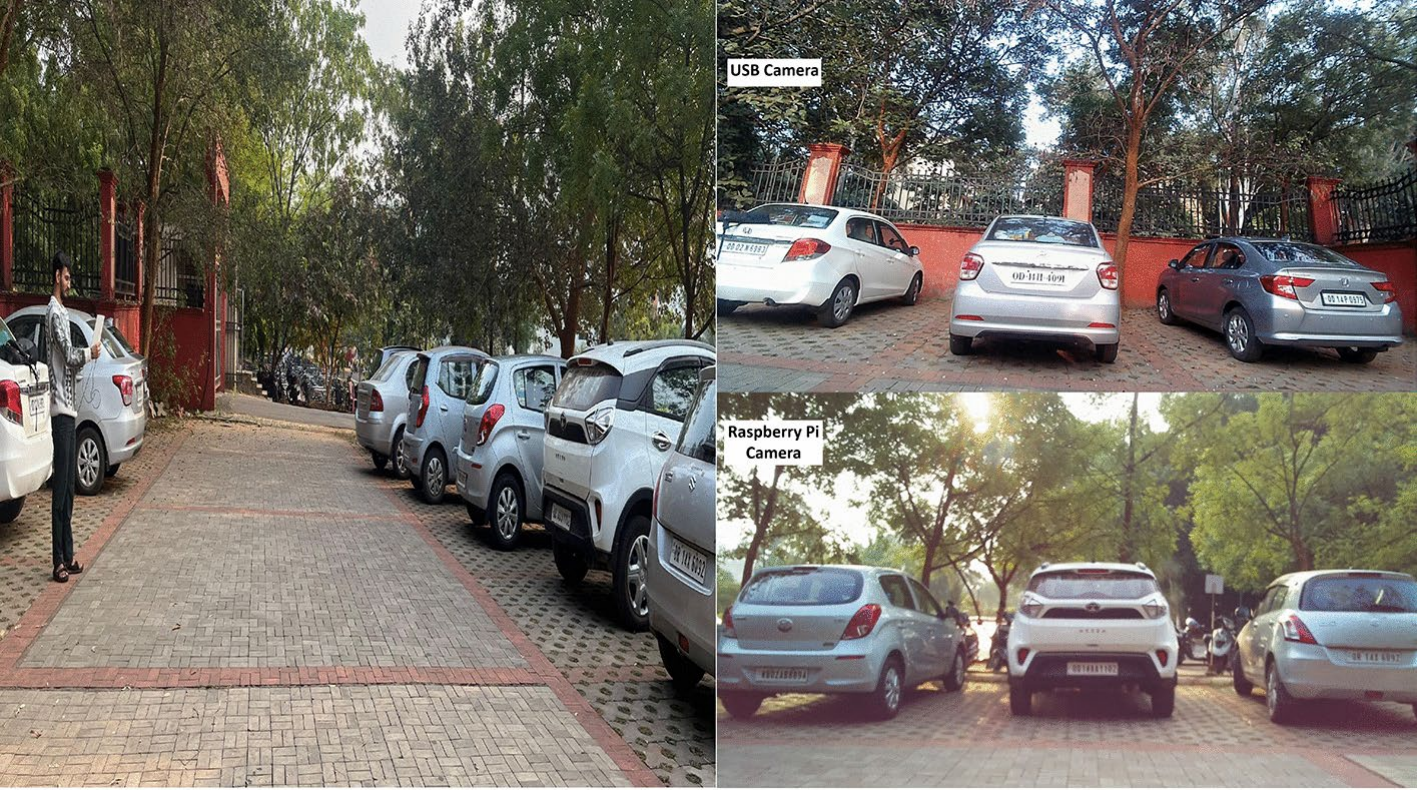
Experimental setup for a Raspberry Pi 4 Model B with Pi camera and USB camera.

The proposed research consists of the following modules:

- Vehicle recognition system (VRS) at the entrance and exit: The vehicle recognition system (VRS) at entry allows the vehicles to enter inside depending on the availability of the free slots inside the lot. The authors of^5^ have suggested an automatic gate open system of the parking lot concerning the availability of the parking slot inside. In the current work, the camera at the entry and exit points in Fig. 1 records the license plate and the time of entry or exit to measure the parking duration. The Raspberry Pi(s) inside the parking lot work as the edge devices and are used to inform the display at the entry about the number of empty slots and accordingly vehicles are allowed inside. Similarly, the camera at the exit will detect the vehicle leaving the lot and update the database accordingly.
- Vehicle parking detection system (VPDS): The vehicle parking detection system (VPDS) shown in Fig. 2 uses an IR sensor along with an ESP32 module and battery at each slot for vehicle detection. Upon arrival of a vehicle inside the parking slot, the IR sensor detects it and the state is communicated to the Raspberry Pi via the ESP32 microcontroller. Four nos of cameras are connected to the microcontroller (Raspberry Pi 4 Model B) by using the USB and the Pi slot to capture multiple vehicles at a time. By using an appropriate computer vision-based algorithm, multiple vehicles in different frames will be captured and the license plates will be detected. Slot-coordinate matching ensures accurate parking assignment, with dual verification from IR sensors to enhance detection accuracy while reducing hardware costs.
  As experimented in Fig. 3 multiple vehicles have been captured by using one Pi camera and one USB camera connected to the Raspberry Pi 4 Model B microcontroller. The details of the license plate extraction by the slot and frame matching algorithm are explained later in this article.
- Smart metering display (SMD) unit: The smart metering display (SMD) unit at the exit in Fig. 1 measures the occupancy duration using data from IR sensors and the camera. Fare details are displayed for customer convenience, supporting the revenue generation of the parking operators. When the IR sensor detects a vehicle in the defined range, it captures the license plate and matches the vehicle number read with the database. It fetches the total Fare stored in the database and displays it at the exit upon finding the match. At the same time, the slot counter is decremented by one for occupied slots and incremented by one for available slots.

### Proposed methodology

**Fig. 4:**
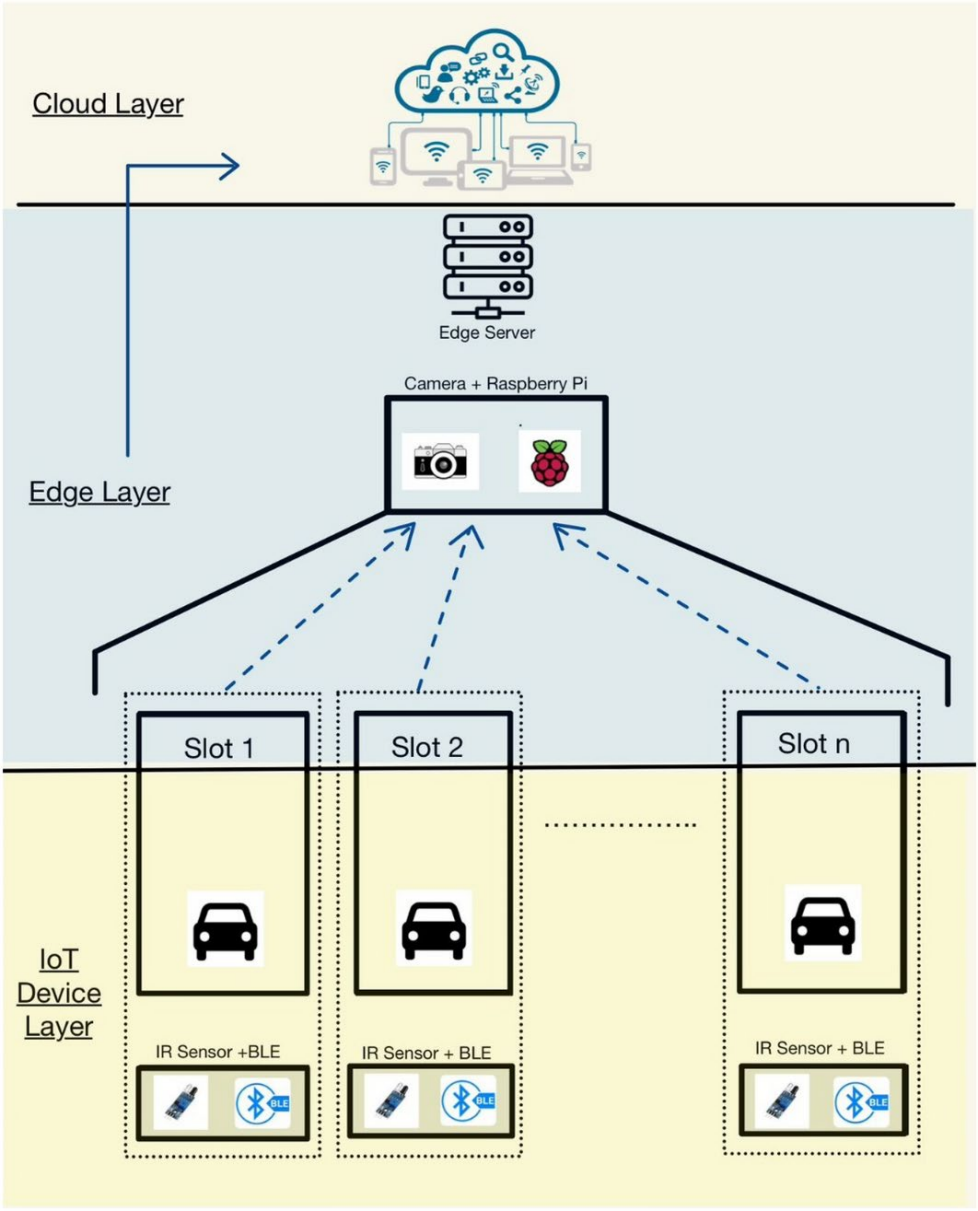
Proposed system architecture for vehicle detection and monitoring setup.

An overall architecture of the VPDS and multiple license plate recognition through a single capture at the edge layer is depicted in Fig. 4.

**Fig. 5:**
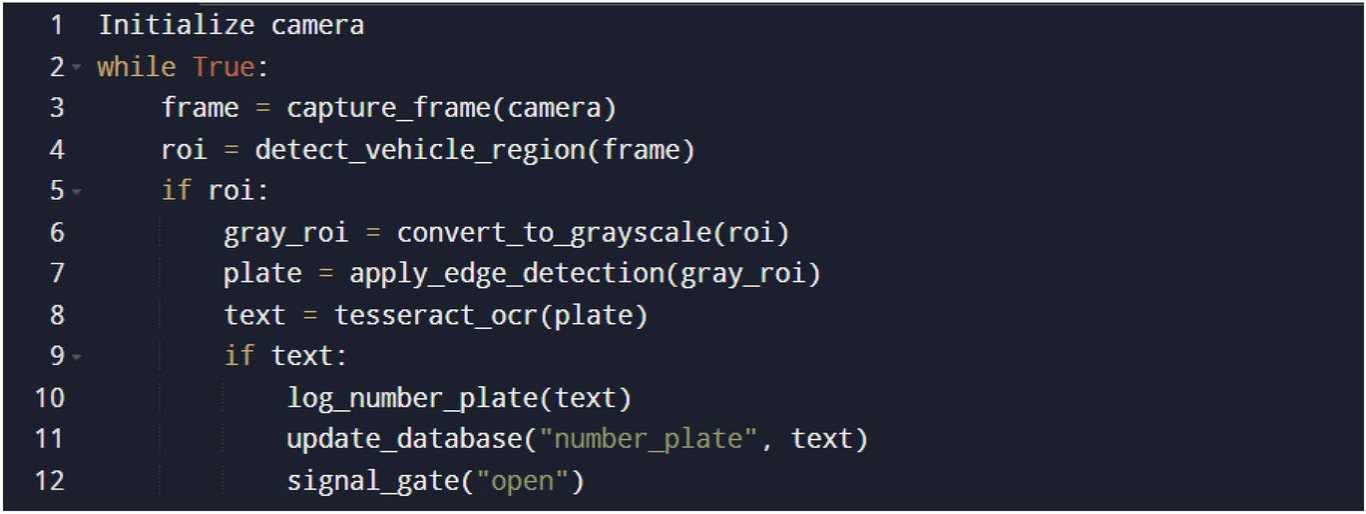
Pseudo code for working of the vehicle recognition system.

When the vehicle enters the parking lot through the Entry gate as shown in Fig. 1, it is detected by the IR sensor at the entry gate which activates the camera in return. The camera then captures the vehicle’s license plate as per the procedure represented in Fig. 5.

As the vehicle is recognised by the camera, the parking duration measurement starts for the vehicle. A similar algorithm is executed at the exit gate to know the exit of the vehicle. Advanced image processing techniques are then applied to the captured image to detect the vehicle’s frame and identify its colour. Using Gaussian Blurring and Thresholding, noise is reduced and the image is segmented for clear vehicle boundaries. Following this, contouring is used to localize and draw a bounding box around the license plate. Once the vehicle’s frame and license plate are isolated, Optical Character Recognition (OCR) is employed to extract the license plate text from the bounding box.

- YOLO detects parked vehicles, matching their coordinates with slot data. Verified occupancy triggers license plate capture and cross-referencing with the database to start the timer and fare calculation.
- The slot counter updates automatically, displaying availability at the parking entry.
- Upon exit, metering stops, and the fare and vehicle details are stored in the database for billing and records.

When a parked vehicle is detected by the camera, the system cross-references the detected coordinates with predefined parking slot locations, verifying occupancy. After successful verification, the license plate of the vehicle is captured using Optical Character Recognition (OCR) and matched with the vehicle database, enabling accurate vehicle tracking and automated fare calculation.

**Fig. 6:**
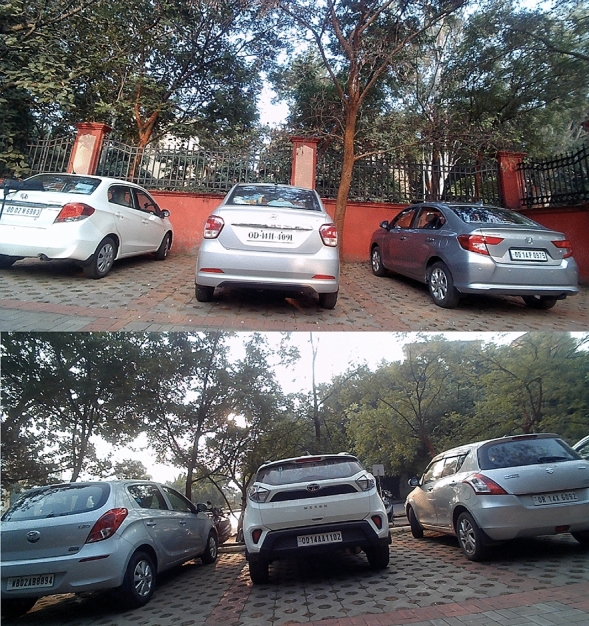
Images in different positions of the Sun by the USB camera connected to Raspberry Pi.

**Fig. 7:**
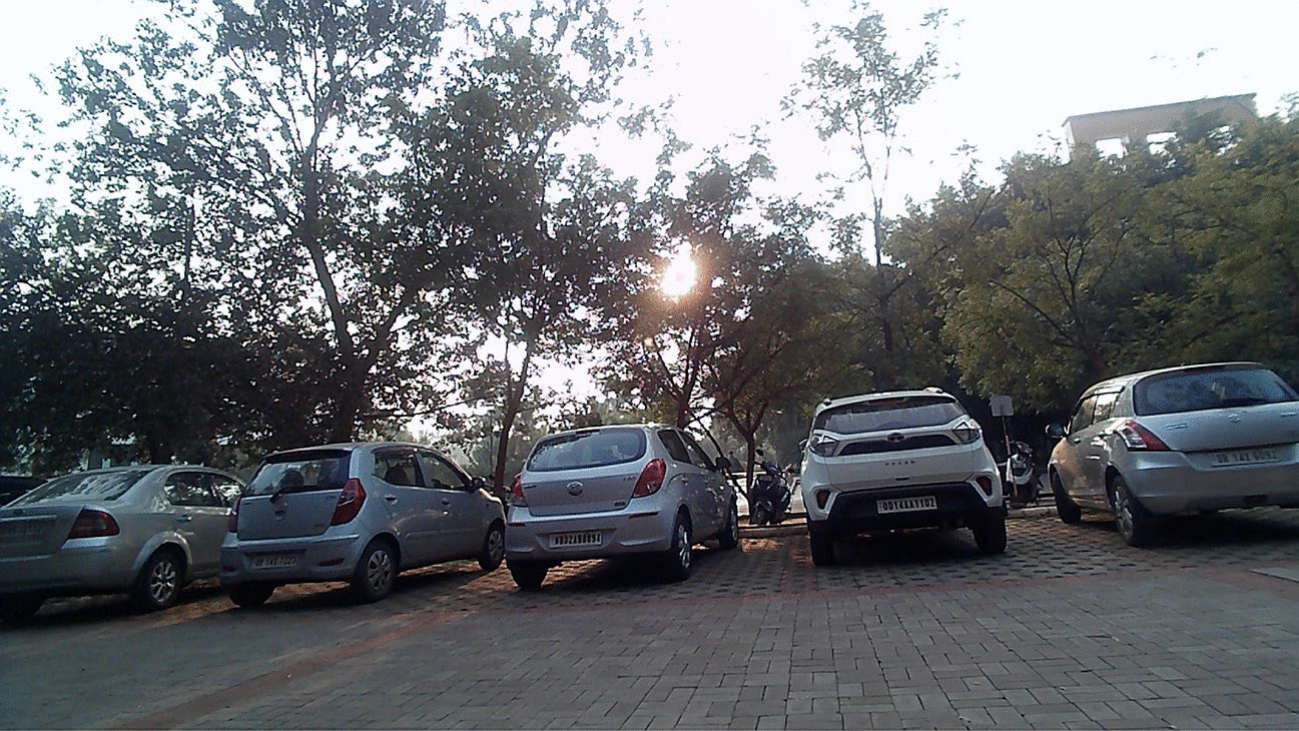
Image captured in USB camera facing towards sun.

Figure 6 shows the experimental images captured by the USB camera in two opposite parking lanes, one facing towards the sun and the other in the opposite direction of the sun. The novelty of the research lies in extracting license plates of individual vehicles from a single frame. It can be understood that the Raspberry Pi microcontroller while connected to four different cameras at the time can capture a total of four images having multiple slots in each image. By using a USB camera in the experiment, one frame can capture 5 parking slots as shown in Fig. 7.

Therefore, depending on the positioning of the camera and the wide angle used by the camera, multiple parking slots can be captured in one frame and the same extraction algorithm can be used for license plate recognition.

**Fig. 8:**
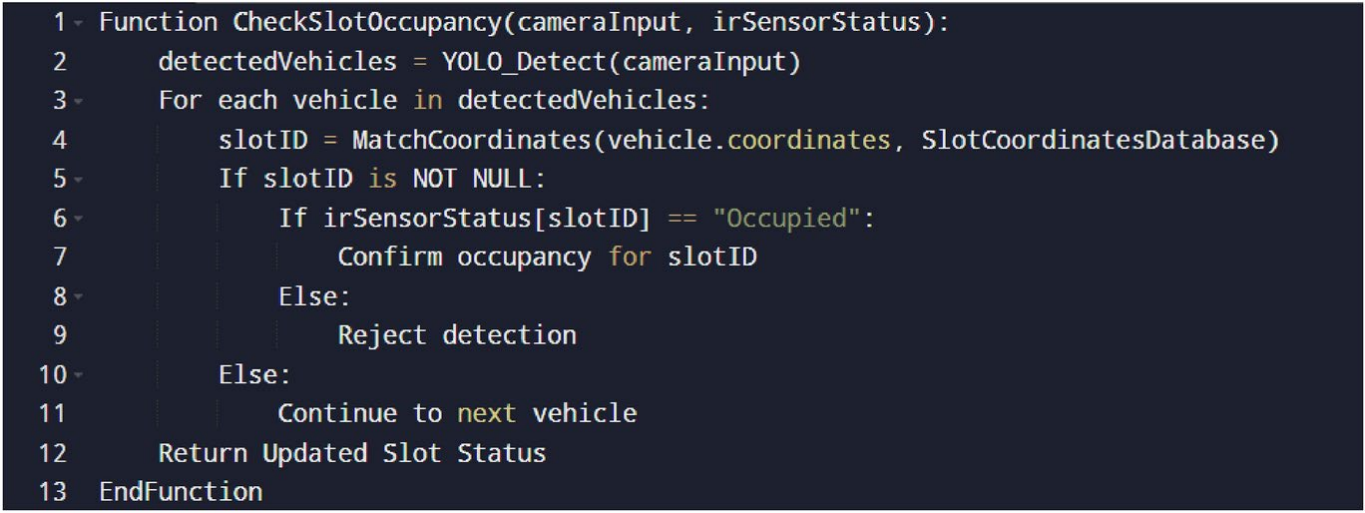
Vehicle coordinates matching algorithm.

**Fig. 9:**
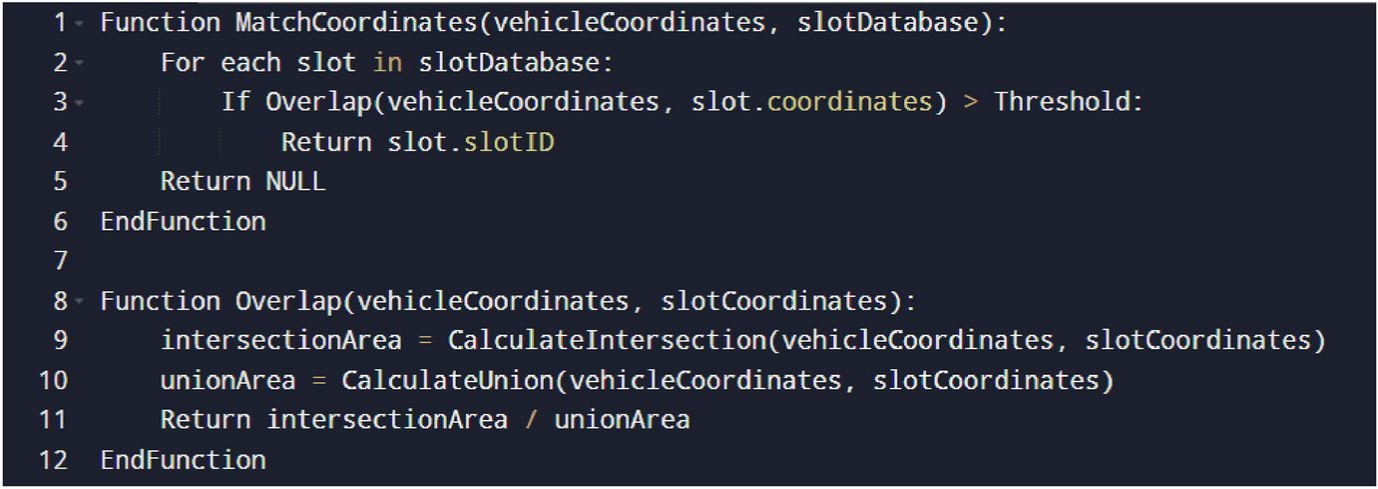
Vehicle validation with IR sensors.

**Fig. 10:**
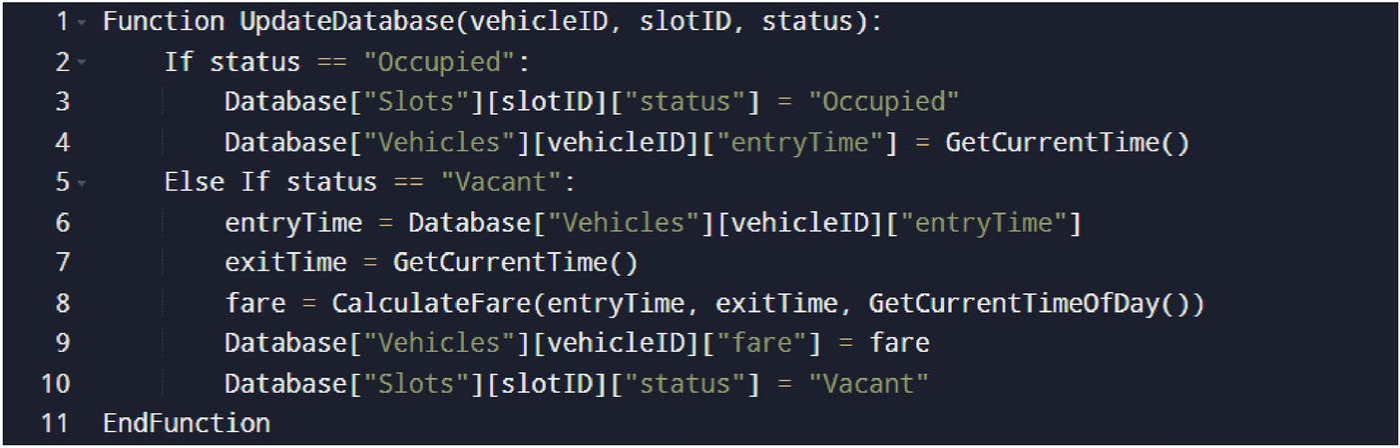
Database updation for slot occupancy and fare calculation.

The following presents the pseudo-codes for matching vehicle coordinates with parking slots (Fig. 8), validating the detected vehicle with IR sensors (Fig. 9) and the corresponding database updation (Fig. 10):

- Vehicle and parking slot coordinates matching:
- Slot coordinates and unique IR ID matching:
- Database Updation Methodology:

### Vehicle identification from the camera feed

**Fig. 11:**
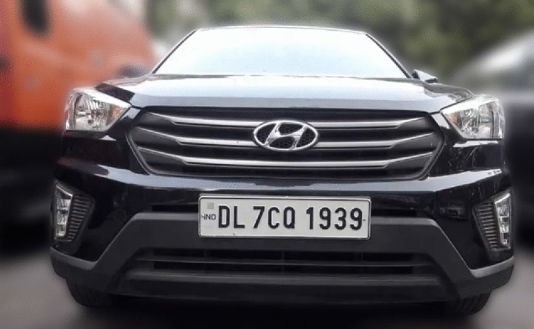
Background noise reduced using Gaussian Blurring technique in the proposed integrated model.

Gaussian Blurring is applied to minimize noise and smooth the camera image. The process of vehicle identification from the camera feed begins with analysing the video feed and using the Gaussian blurring technique focusing on a threshold value of vehicles and license plates based on the still image contrast. The result of applying this technique is depicted in Fig. 11.

**Fig. 12:**
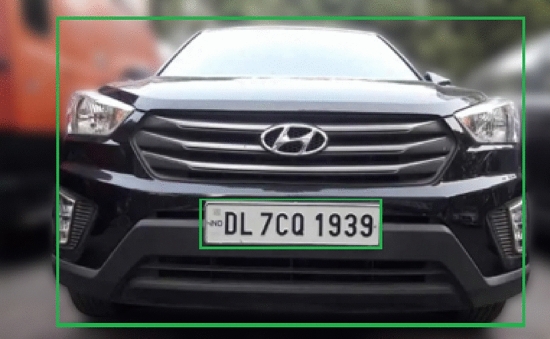
Vehicle and license plate identification with bounding boxes in the proposed model.

A grid-based detection approach is applied which identifies the vehicle by placing an abounding box around it and then isolating the license plate with a secondary boundary box specifying its exact coordinates. This dual detection method ensures the precise identification of vehicles for subsequent processing. The result is shown in Fig. 12

#### Gaussian blurring technique

In this proposed work, Gaussian blurring is applied to the HSV (Hue, Saturation, Value) image to reduce noise and smoothen the image. This is achieved by convolving the image with a Gaussian kernel. From the study explained thoroughly in Kostková et al.^7^ mathematically, the Gaussian blur operation can be represented as:

1 $$\begin{aligned} G(x, y) {=} \frac{1}{2\pi \sigma ^2} \cdot e^{-\frac{x^2 + y^2}{2\sigma ^2}} \end{aligned}$$

where:

1. $G(x, y)$ is the Gaussian kernel at position $(x, y)$.
2. $\sigma$ is the standard deviation of the Gaussian distribution.
3. $e$ is the base of the natural logarithm.

The Gaussian blur operation is performed by averaging the pixels in the vicinity of each pixel, giving more weight to the central pixels. This effectively reduces high-frequency noise in the image.

In the proposed implementation, the technique is applied to the HSV image using a kernel size of $(11, 11)$ and a standard deviation of 0, as shown in Fig. 11. Adjusting the kernel size or the standard deviation can alter the blurring effect. Smaller kernel sizes or more minor standard deviations produce lighter blurs, while larger values create stronger blurs.

#### Thresholding

Thresholding is a fundamental image processing technique that converts grayscale images into binary ones by categorizing pixels as either black (0) or white (255) based on a predetermined threshold value. From a study explained thoroughly—mathematically, the thresholding operation can be represented as:

2 $$\begin{aligned} \text {Binary}(x, y) = {\left\{ \begin{array}{ll} 1, & \text {if } \text {Gray}(x, y) > \text {Threshold} \\ 0, & \text {otherwise} \end{array}\right. } \end{aligned}$$

where:

1. $\text {Binary}(x, y)$ represents the binary output image at position $(x, y)$.
2. $\text {Gray}(x, y)$ is the grayscale value of the input image at position $(x, y)$.
3. $\text {Threshold}$ is the predefined threshold value.

Here, thresholding is applied after converting the frame to the HSV colour space. The ‘cv2.inRange()’ function creates a binary mask by isolating specific colours within the specified range defined by ‘lower_color’ and ‘upper_color’. This binary mask aids in identifying edges through subsequent contour detection using the Canny edge detection algorithm (‘cv2.Canny()’).

#### YOLO (You Only Look Once) method

YOLO is an advanced, grid-based object detection algorithm known for its speed and accuracy in detecting and localizing objects within an image. In this proposed model, YOLO is utilized to detect both the vehicle boundary and the license plate within the processed image, which has undergone preliminary enhancement techniques, such as Gaussian Blurring and Thresholding.

In YOLO, the input image is divided into an $S \times S$ grid, where each grid cell is responsible for predicting bounding boxes and class probabilities for the objects within it. Given an input frame, YOLO processes the entire image at once, predicting bounding boxes and their associated confidence scores in a single pass through the network.

The mathematical approach for YOLO detection can be described as follows:

1. Bounding box prediction: Each grid cell predicts $B$ bounding boxes. A bounding box is defined by four parameters $(x, y, w, h)$:
  - $(x, y)$: The coordinates representing the center of the bounding box.
  - $w$: The width of the bounding box relative to the entire image.
  - $h$: The height of the bounding box relative to the entire image.
2. Confidence score: Each bounding box has an associated confidence score that reflects the likelihood of containing an object and the accuracy of the bounding box coordinates:
  3 $$\begin{aligned} \text {Confidence Score} = P(\text {object}) \times \text {IOU}_{\text {pred}}^{\text {truth}} \end{aligned}$$
  where $P(\text {object})$ is the probability of an object being in the bounding box, and $\text {IOU}_{\text {pred}}^{\text {truth}}$ is the Intersection over Union between the predicted bounding box and the ground truth.
3. Class prediction: Each grid cell also predicts $C$ class probabilities for the presence of an object within the cell:
  4 $$\begin{aligned} \text {Class Probability} = P(\text {class} | \text {object}) \end{aligned}$$
  YOLO calculates the final score for each bounding box by multiplying the confidence score with the class probabilities.

In this work, YOLO first identifies a bounding box around the vehicle boundary, marking this area as the Region of Interest (ROI) for the next detection stage. The algorithm then re-evaluates the ROI for a finer license plate detection, creating a nested bounding box within the vehicle boundary as shown in Fig. 12. This dual-stage detection allows for targeted processing, ensuring that only relevant portions of the frame are considered, reducing unnecessary computation and enhancing accuracy.

The mathematical representation of this grid-based approach is:

5 $$\begin{aligned} \text {Output}(i, j) = {\left\{ \begin{array}{ll} (x, y, w, h, \text {Confidence}, P(\text {class} | \text {object})), & \text {if object detected in grid cell} \\ 0, & \text {otherwise} \end{array}\right. } \end{aligned}$$

where $(i, j)$ represents a specific grid cell.

After identifying the vehicle boundary with a bounding box, a nested bounding box is drawn within it, targeting the license plate based on additional YOLO predictions focused within the ROI. This hierarchical detection technique, combined with earlier pre-processing steps (Thresholding, Gaussian Blurring, etc.), produces robust results in detecting both the vehicle and its license plate, facilitating further stages like Optical Character Recognition (OCR) for license plate text extraction.

### License plate recognition

A study by Du et al.^21^ reviews Automatic License Plate Recognition (ALPR) technology, outlining its applications, operational principles, and challenges. It categorizes ALPR techniques based on features used and compares them regarding accuracy and inference. However, it lacks detailed discussions on limitations and recent advancements and could benefit from practical examples to illustrate effectiveness.

This model overcomes these shortcomings in traditional ALPR techniques by effectively handling diverse environmental conditions, accommodating license plates from different regions, enhancing image quality, and enabling real-time processing.

This section covers the crucial step of extracting text information from images using Optical Character Recognition (OCR). The captured image is processed using Grayscale Conversion and Noise Reduction techniques to enhance the text. The OCR software (Tesseract) recognises and extracts the text. Tesseract analyzes the image, identifies character patterns, and converts them into machine-readable text.

#### Gray scale conversion

Converting to a grayscale image is very important in pre-processing the images before they are analyzed or used in computer vision. This entails changing an image’s initial colour representation. Usually, RGB (red, green, and blue) is used in grayscale, where each pixel value reflects brightness or luminance. From the study explained thoroughly in Saravanan et al.^22^ mathematically, it can be expressed as:

6 $$\begin{aligned} Y = 0.299 \times R + 0.587 \times G + 0.114 \times B \end{aligned}$$

where:

1. $R, G, B$ represent red, green and blue colour planes for the original image.
2. $Y$ denotes the resulting intensity value in grayscale.

**Fig. 13:**
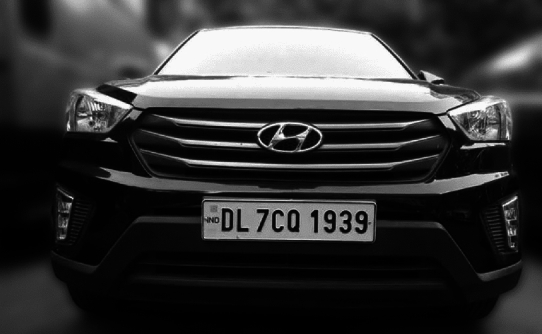
Grayscale conversion of an RGB image for text extraction.

In the case of text extraction, converting images into grayscale simplifies them to the extent that one channel does not retain any essential information required by text but has reduced computational load, which can be understood from Fig. 13. By expressing an image using its intensity values, grayscale conversion brings out more vivid details concerning texts and contrasts, making it easier to see words against other parts of a document. This makes things simple for subsequent algorithms to recognize such texts because instead of dealing with each transition’s changes in intensity related to each character’s font style.

#### Noise reduction

Noise in images means random changes in pixel values, which may lead to poor image quality and disrupt text extraction accuracy. Reducing noise is essential because it helps to reveal details hidden within an image. One of the most frequently employed methods for reducing noise is Gaussian Blur, a spatial filter that smooths the image by minimizing the effect of high-frequency noise.

In simple terms, Gaussian Blur works by summing weighted averages for each pixel based on values obtained through a Gaussian function surrounding a given pixel. This operation can be written as:

7 $$\begin{aligned} \text {Blur}(x, y) = \sum _{i=-k}^{k} \sum _{j=-k}^{k} \text {Pixel}(x+i, y+j) \cdot \text {Kernel}(i, j) \end{aligned}$$

where:

1. $\text {Blur}(x, y)$, is the resulting intensity value at (x,y).
2. $\text {Pixel}(x+i, y+j)$, indicates the intensity value of neighbouring pixels relative to an offset (i,j).
3. $K(i,j)$, represents the coefficient of Gaussian kernel at position (i,j).
4. k stands for kernel size.

**Fig. 14:**
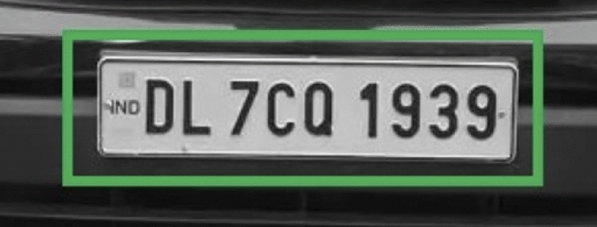
Reducing noise by subtracting the background and reducing the image size, emphasising the License Plate.

The process significantly removes high-frequency noise while preserving vital features like edges and text, thus smoothing the image. The result is reduced noise level and improved clarity, making extracting meaningful text more precisely easier, as shown in Fig. 14. Gaussian Blur is a fundamental noise reduction technique that plays a crucial role in enhancing the accuracy of text extraction algorithms by mitigating the impact of noise in the input image.

#### Tesseract

Tesseract, an open-source Optical Character Recognition (OCR) engine developed by Google, employs advanced algorithms to extract text from images. Its functionality can be mathematically represented as:

8 $$\begin{aligned} \text {Text}(x, y) = \text {Tesseract}(I(x, y)) \end{aligned}$$

where:

1. $\text {Text}(x, y)$ represents the recognized text at position $(x, y)$ in the image.
2. $I(x, y)$ denotes the intensity or pixel value at position $(x, y)$ in the input image.

Tesseract analyzes the structural features of text within the image, recognizing patterns and converting them into machine-readable text. This process involves sophisticated pattern recognition algorithms that discern text elements from background noise and other visual distractions.

**Fig. 15:**
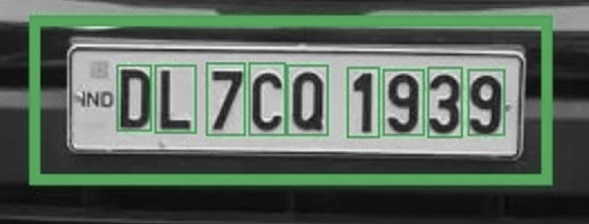
Individual character recognition by creating bounding boxes separately around each character.

Due to its robust design, Tesseract can accurately detect and extract text from images with complex backgrounds or distortion, as shown in Fig. 15. Its versatility and reliability make it a valuable tool for various text recognition applications, from document scanning to image-based data extraction in various industries.

### Smart metering

Smart metering is the process of measuring the parking duration of a vehicle from its entry to the exit. The fare calculation system is designed to compute charges based on the vehicle’s occupancy duration in the parking slot. The formula used is:

9 $$\begin{aligned} \text {Total} \ \text {Fare} = \text {Occupancy}\ \text {Duration} * \text {Cost}\ \text {per}\ \text {Second} \end{aligned}$$

**Fig. 16:**
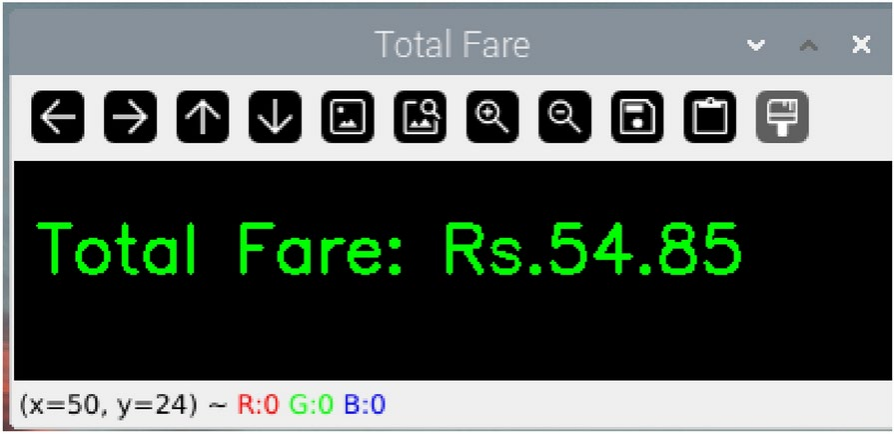
Smart metering device displaying the total fare at parking exit.

Occupancy duration is recorded with accurate timestamps captured through sensor and image processing technologies. The cost per second is a predefined rate used to compute the total charges automatically. The system supports a dynamic pricing structure that adjusts rates based on peak and off-peak hours to enhance efficiency, which is displayed on the smart metering device (Fig. 16). This ensures fair billing while optimizing resource usage. By automating the fare calculation process, the system minimizes human error, providing a reliable and scalable billing solution to the parking operator.

### Database integration

**Fig. 17:**
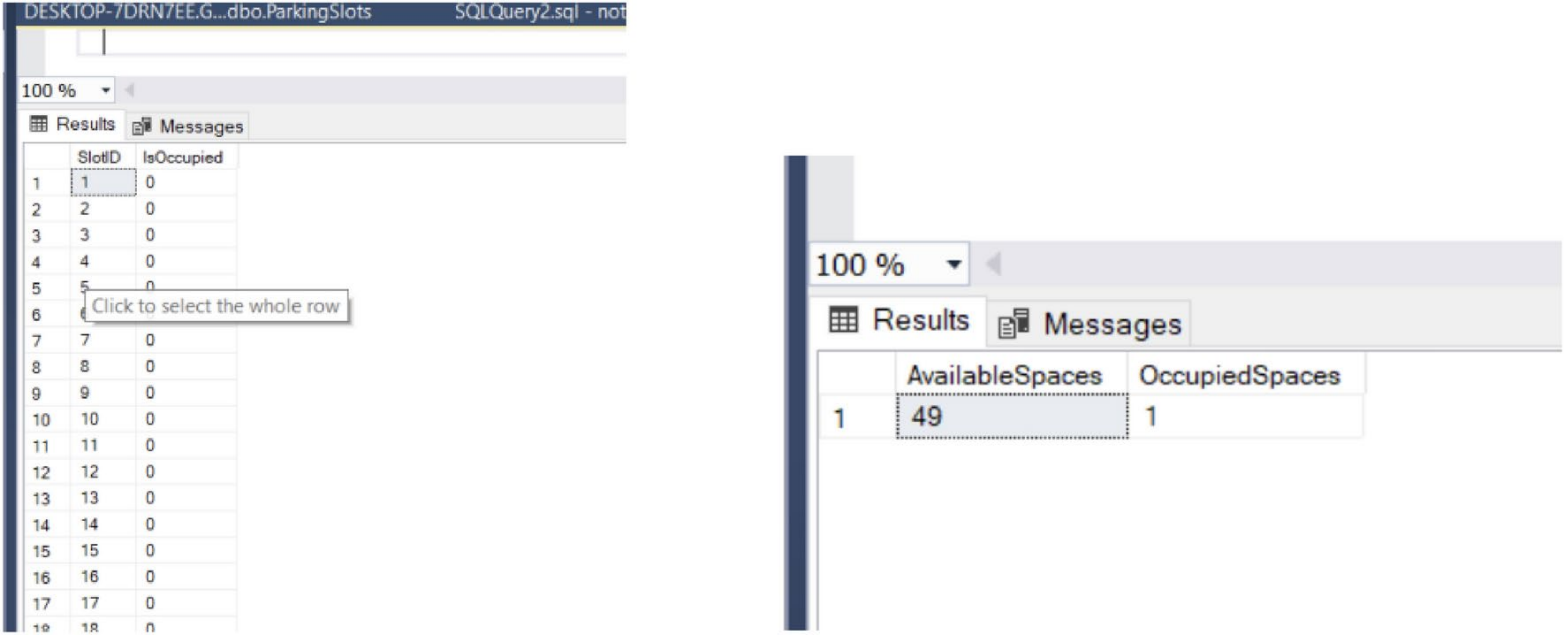
Database of Parking with 50 Test slots with a unique SlotID dedicated to each slot and slots counter for the number of available and occupied parking slots.

The integration of a centralized database system, implemented using Microsoft SQL Server Management Studio (SSMS) and SQLite, enhances the efficiency and reliability of the proposed parking management system as shown in Fig. 17. The database is designed to store critical information such as vehicle details, slot coordinates, IR sensor IDs, occupancy status, and fare calculation data. This robust setup ensures seamless data storage, retrieval, and management, supporting the system’s core functionalities.

**Fig. 18:**
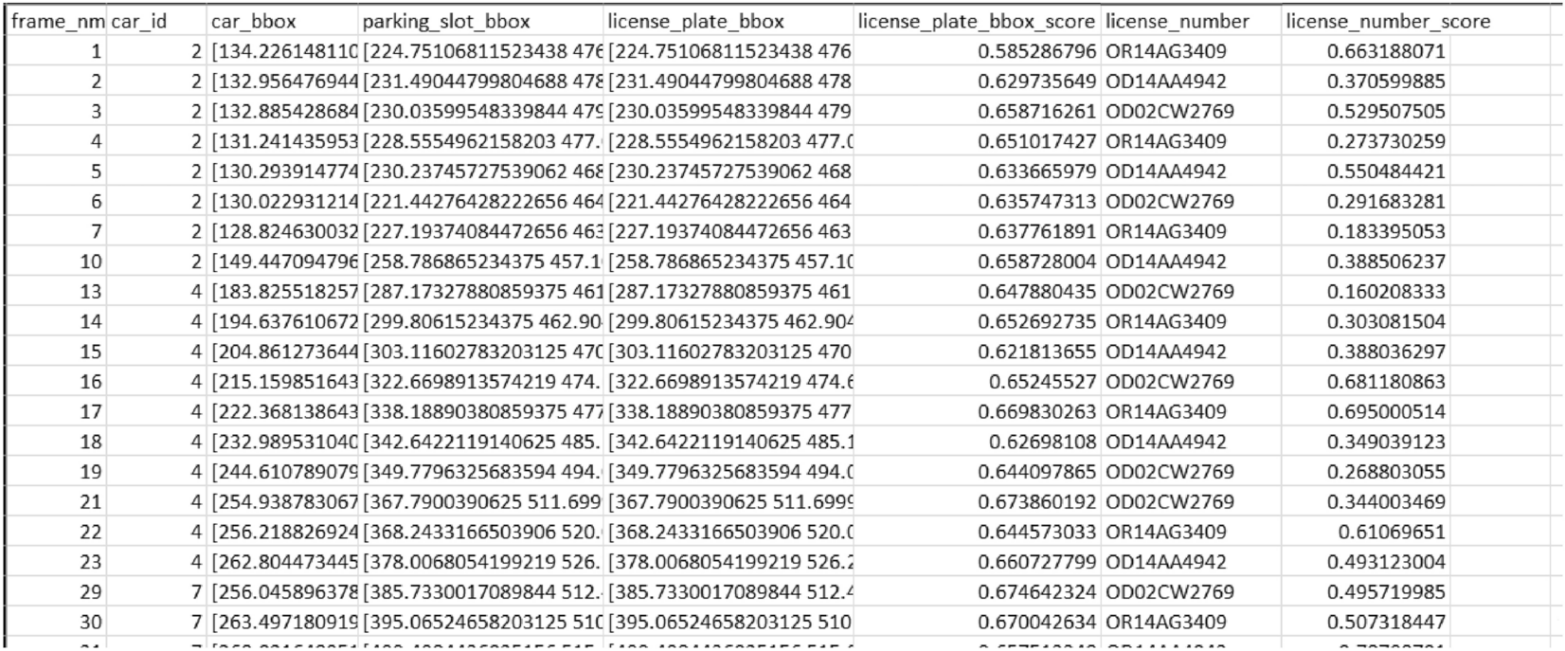
Exported CSV file of the processed database.

Key features of database integration include:

- Slot and vehicle matching: The database facilitates the precise association of detected vehicles with predefined slot coordinates as shown in Fig. 18 using a combination of YOLO-based detection and IR sensor ID confirmation. When the column values of D and E match, the parking of a vehicle is verified.
- Real-Time Slot Monitoring: The system continuously updates the occupancy status of parking slots, providing real-time data for slot availability displayed at the entrance. This reduces congestion and optimizes parking space utilization.
- Fare Calculation: Occupancy duration and dynamic pricing details are stored in the database, enabling automated, accurate billing. Each vehicle’s parking history and fare details are maintained for record-keeping and audit purposes.
- Admin Operations: Administrators can use SSMS to interact with the database, updating or retrieving data as needed. This simplifies operations such as adjusting pricing models, tracking resource usage, and managing slot assignments.

The integration of SSMS also strengthens data security and ensures that the system can scale to accommodate larger parking lots or advanced features like predictive analytics and customized user preferences. By consolidating key functionalities into a centralized database, the system ensures smooth operations, enhances resource allocation, and provides a seamless parking experience for both administrators and users.

## Results and analysis

### Vehicle identification and text recognition

The vehicle identification and text recognition modules have shown remarkable accuracy in identifying vehicles in various real-world conditions. However, to ensure the robustness of the system under different scenarios, additional experiments were conducted under a range of lighting conditions, plate orientations, and varying distances. These extended tests have provided more representative results of the system’s performance in practical parking environments.

**Fig. 19:**
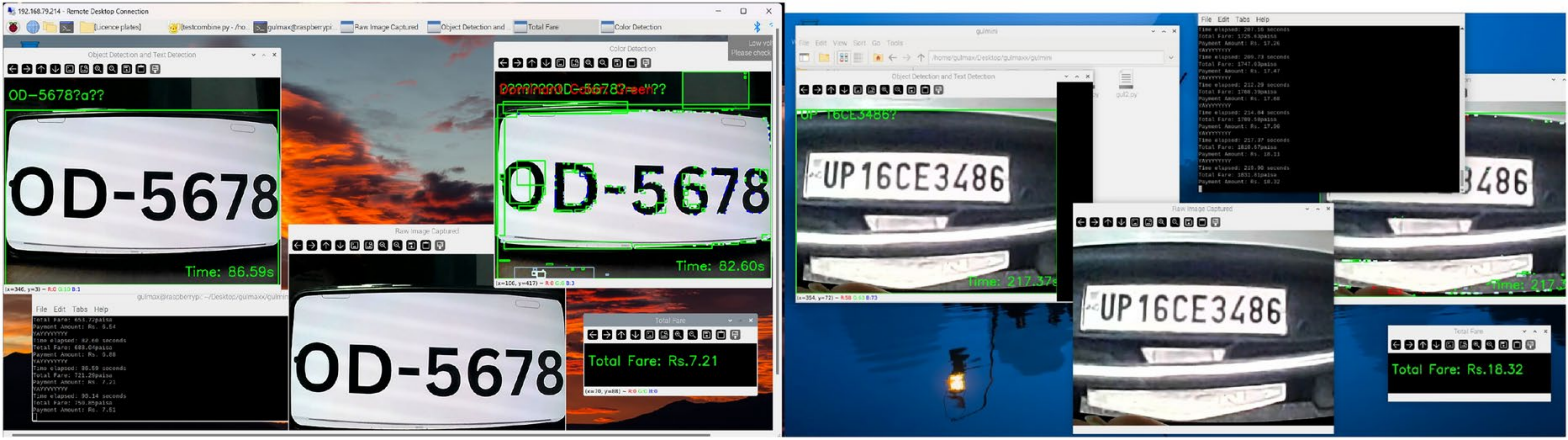
Results for high and low light conditions.

- Lighting conditions: The system was tested under direct sunlight as in Fig. 6, low-light environments, and artificial lighting typical in parking lots. The system maintained high accuracy (up to 95%) in daylight conditions and still performed effectively (90%) under low-light settings, ensuring its adaptability for 24/7 operations. Figure 19 showcases the results for two different light conditions, including vehicle identification, text recognition, and fare calculation, demonstrating the robust performance and integration of the proposed system.

**Fig. 20:**
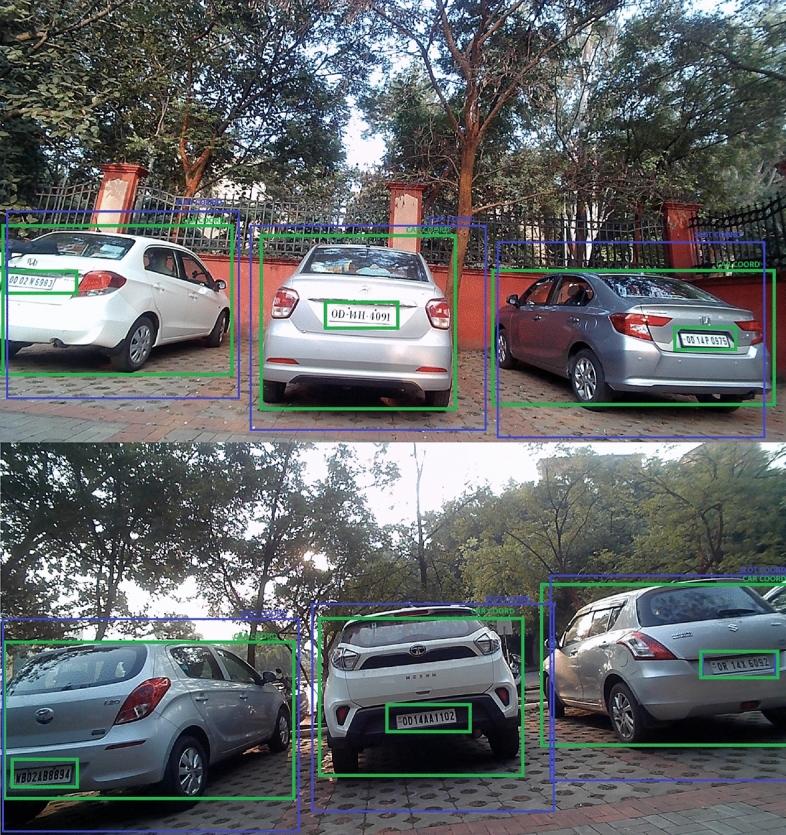
Implementation results validating coordinates matching of the vehicle at a defined distance.

These experiments validate the proposed dual-verification mechanism combining camera-based vehicle identification with IR sensor-based detection. Figure 20 demonstrates the implementation of the vehicle coordinate matching system. In the visual, the blue boxes represent the predefined slot coordinates within the parking area, while the green boxes highlight the vehicle’s detected coordinates. The validated coordinates are then written to the database for precise slot tracking.

To validate the accuracy of the proposed model, it was evaluated across 100 tests. These tests included license plates(individual and multiple number plates in one frame) from various vehicles under multiple conditions. As shown in Table 1, the proposed system successfully matched the correct license plate number in 88.9% of the 100 test cases. While few test cases failed, the overall performance confirms the system’s reliability for vehicle identification.

**Table 1:** Test case comparison depicting the original license plate number and the result obtained by the proposed model.

Lighting	Original number	Model result	Camera position
Low light		MH2D BY 4465	Angled
Bright light		GL 395 X	Angled
Low light		K1 1O AW PI11	Zero angle
Bright light		UK O4 AC 8)I8	Angled
Low light		KA 17 MA D825	Angled
Bright light		OD 14 AA 4942	Angled
Bright light		OO 14 AG 3409	Zero angle
Bright light		OD 02 CW 2769	Zero angle

### Comparison of the device components of proposed model with existing models

A detailed comparison of the hardware and infrastructure setup is provided in Table 2. For a dozen parking slots inside a parking lot, it provides the list of the total no of hardware required. It is evident that by leveraging the IoT technology multiple cameras can be connected to one Raspberry Pi in different directions to capture the image of multiple vehicles in a single frame. Further, by using the clot matching mechanism individual license plates can be extracted from the same frame as proposed. Out of all the IoT components used by the researchers, Raspberry Pi is the pricy one. The authors of^2^ are using a maximum no. of Pi controllers enhancing the cost of the hardware and the infrastructure setup. Similarly, though the authors of^4^ replace low-cost ESP32 microcontrollers, use of single frame cameras for individual parking slots enhances the cost associated. The proposed research utilises the least number of Raspberry Pi and the cameras for the same operating the same number of parking slots.

**Table 2:** Comparison for the hardware and infrastructure setup for 12 parking slots.

Approach	Raspberry Pi	USB camera	Pi camera	ESP-32	Sensor	Cost
Proposed work	1	3	1	12	12	Low
Ramachandra et al.^4^	0	12	0	12	12	Medium
Michel-Torres et al.^2,3^	12	0	12	0	12	High

## Conclusion and future work

The proposed work marks a significant milestone in developing cutting-edge systems for efficient vehicle identification, parking slot allocation, license plate recognition, and parking fare calculation. The successful integration of IoT, image processing, and database management enables the proposed system with minimal hardware setup and cost. Reducing the number of costly microcontrollers with low cost ESP32 microcontrollers and connecting multiple cameras to the Raspberry Pi is the major focus of the work. Further, enhanced computer vision method to extract individual license plates from a single camera frame with multiple license plates is another milestone achieved. The system’s accuracy and reliability in determining vehicle presence duration and calculating fares demonstrate its vast potential for implementation in various settings, including parking management, toll systems, and traffic monitoring. A key achievement is eliminating manual errors and enhancing billing efficiency through automated fare calculation.

Looking ahead, several opportunities for future enhancements and adaptations emerge. These include capturing multiple license plates with a single camera will be very useful for toll systems where multiple lanes function in parallel at the payment counter. The use of a single camera for the same purpose can reduce the cost of infrastructure setup as well. The same can be also applied to multi-lane highways to monitor vehicle movement. Dynamic pricing models based on demand and traffic conditions could also be implemented. This integrated approach minimizes setup costs while maintaining high detection accuracy, making it an efficient solution for modern parking management systems.

## Data Availability

The datasets used and analysed during the current study are available from the corresponding author upon reasonable request.
